# Access to orphan drugs in adults with inherited metabolic diseases in Switzerland: a single-center retrospective cohort study

**DOI:** 10.1186/s13023-026-04367-6

**Published:** 2026-05-18

**Authors:** Giovanni De Antoni, Camille Kumps, Jérôme Berger, Jasmin Barman-Aksözen, Christel Tran

**Affiliations:** 1 Division of Genetic Medicine, University of Lausanne and University Hospital of Lausanne, Lausanne, Switzerland; 2 Community Pharmacy, Center for Primary Care and Public Health (Unisanté), University of Lausanne, Lausanne, Switzerland; 3 Center for Research and Innovation in Clinical Pharmaceutical Sciences, Lausanne University Hospital and University of Lausanne, Lausanne, Switzerland; 4 School of Pharmaceutical Sciences, University of Geneva, Geneva, Switzerland; 5 Institute of Pharmaceutical Sciences of Western Switzerland, University of Geneva and University of Lausanne, Geneva and Lausanne, Switzerland; 6 Institute of Laboratory Medicine, Municipal Hospital Zurich Triemli, Zurich, Switzerland; 7 Division of Metabolism and Children’s Research Center, University Children’s Hospital, Zurich, Switzerland; 8 University Research Priority Program “ITINERARE – Innovative Therapies in Rare Diseases”, University of Zurich, Zurich, Switzerland

**Keywords:** Orphan drugs, Inherited metabolic diseases, Therapies

## Abstract

**Background:**

Orphan drugs (ODs) are increasingly used to treat rare diseases, including inherited metabolic diseases (IMDs), but real-world access remains insufficiently characterized. This study aimed to evaluate access to ODs in adults with IMDs treated at a Swiss reference center and to analyze the associated regulatory and reimbursement frameworks.

**Methods:**

In this retrospective, single-center study conducted between 2017 and 2022, we included all adult patients with a confirmed biochemical and/or genetic diagnosis of an IMD who received an OD. The primary outcome was the proportion of patients receiving OD therapy. Secondary outcomes included the types of ODs used, reimbursement procedures, time from treatment indication to treatment initiation, timelines in Swiss marketing authorization (MA) and reimbursement compared with international benchmarks.

**Results:**

Of 190 patients followed, 41 (21.6%) had an indication for OD therapy and 39 (20.5%) received treatment (median age 30.3 years, range 19–65). Seventeen different ODs were prescribed. The median time from treatment indication to treatment initiation was 1.0 month [IQR 0.6–2.6] but was longer for drugs without Swiss MA (Art. 71c OAMal: 6.5 months [IQR 2.5–11.9]). Swissmedic MA occurred a median of 1.9 years [IQR 1.5–2.7] after approval by other international regulators. Three patients were the first adults in Switzerland to access novel substances (olipudase alfa, pegvaliase, metreleptin), and one received the newly approved formulation of nitisinone.

**Conclusion:**

In this cohort from a specialized adult metabolic clinic, most patients with an indication for OD therapy accessed treatment. However, administrative burden, fragmented reimbursement procedures, and regulatory delays may still affect timely treatment initiation in certain situations. These findings highlight the need for continued efforts to streamline regulatory and reimbursement pathways and to ensure equitable access to innovative therapies for rare diseases.

**Trial registration:**

ClinicalTrials.gov, NCT05818566, registered 18 April 2023.

**Supplementary Information:**

The online version contains supplementary material available at 10.1186/s13023-026-04367-6.

## Introduction

Inherited metabolic diseases (IMDs) are a heterogeneous group of genetic disorders resulting from enzymes, transport proteins, or organelle dysfunction involved in metabolic pathways affecting protein, fat, carbohydrate, heme, or purine/pyrimidine metabolism. These disorders may lead either to the accumulation of toxic substrates or to deficiency of essential products, frequently causing complex multisystemic clinical presentations [1, 2]. Based on their pathophysiology, IMDs can be grouped into three broad categories: storage disorders, intoxication (small molecule) disorders, and energy defects. Although each IMD is individually rare (affecting fewer than 1 in 2,000 individuals in the European Union), their overall incidence is estimated to exceed 1 in 800 live births. These estimates illustrate that while individually rare, IMDs collectively represent a significant clinical burden in the population [3, 4]. Advances in neonatal screening, diagnostic technologies, and therapies have improved survival and quality of life, while increasing recognition of milder and late-onset phenotypes presenting in adulthood. Consequently, an increasing number of adults require long-term specialized metabolic care [5–7].

The development of novel therapies for IMD, many of which have orphan designation, has improved the care of these patients [8, 9]. An orphan drug (OD) designation provides regulatory incentives for therapies targeting rare diseases. Approval for clinical use requires marketing authorization (MA), granted after evaluation of quality, safety, and efficacy. Approved ODs cover a range of therapeutic approaches, including enzyme replacement therapies, substrate inhibitor, chelator, cofactor supplementation and more recently, gene therapy [10]. OD designation is granted by regulatory authorities, such as the U.S. Food and Drug Administration (FDA) [11] in the U.S., the European Medicines Agency (EMA) [12] in the European Union, the Pharmaceuticals and Medical Devices Agency (PMDA) [13] in Japan or Swissmedic in Switzerland [14]. Criteria typically require that the condition is rare, chronically debilitating, or life-threatening, and that the treatment provides a scientific rationale and potential clinical benefit. However, neither OD designation nor MA guarantees timely patient access. After regulatory approval, access may remain limited due to high costs and country-specific reimbursement processes, leading to disparities both between and within healthcare systems [15–17].

**Fig. 1:**
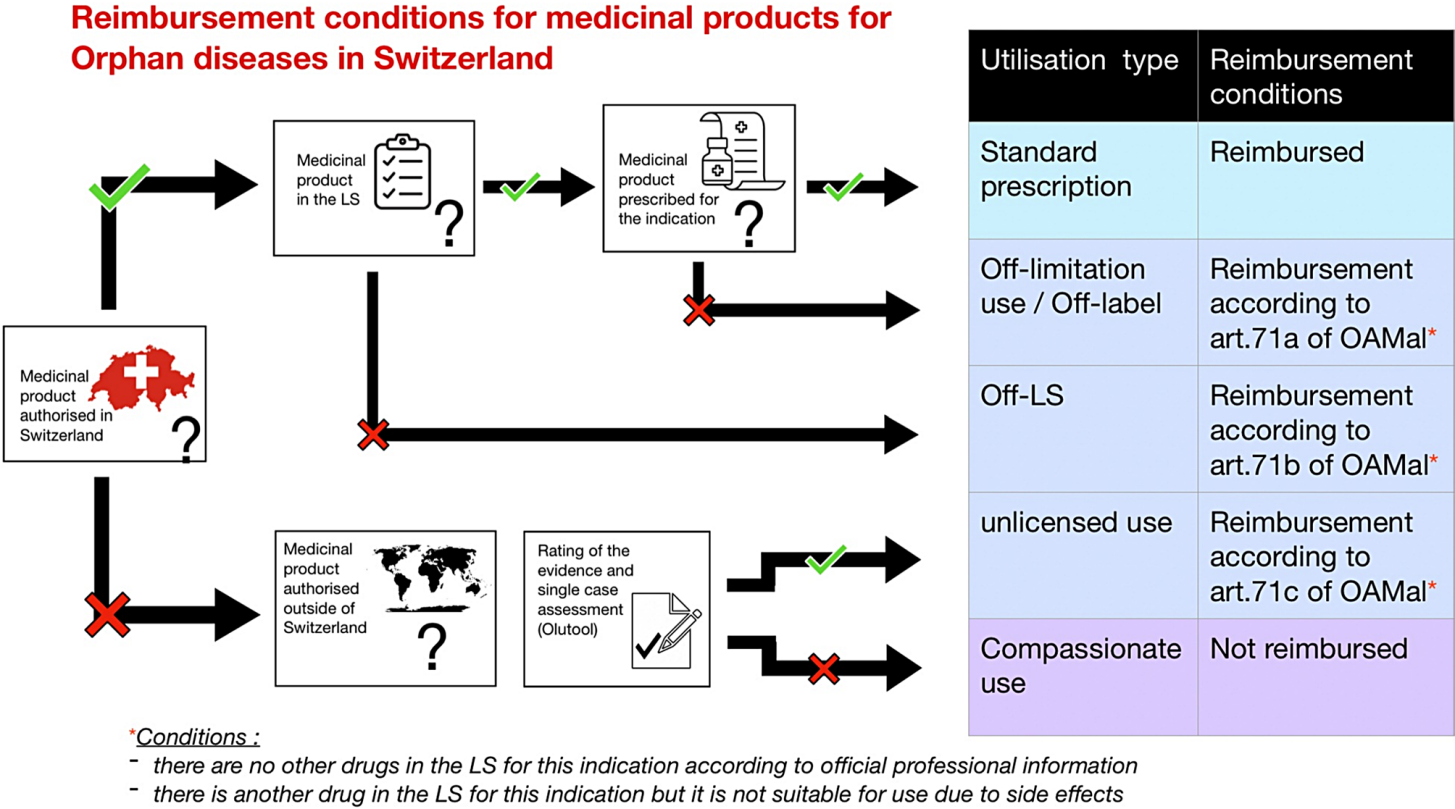
Reimbursement conditions for medicinal products for orphan diseases in Switzerland. Adapted from a scheme developed by the Swiss Cancer League (2020)

In Switzerland, Swissmedic grants both OD designation and MA, which are distinct steps: the former supports development, while the latter allows clinical use. After MA, pharmaceutical companies must apply to the Federal Office of Public Health (FOPH) for inclusion in the List of Pharmaceutical Specialties (LS) [18] or, when applicable, the List for the Treatment of Birth Defects and Congenital Disorders (GGSL/LSIC). These listings ensure reimbursement through mandatory health insurance when used according to the label. However, these procedures are often lengthy, and some companies decline to apply due to administrative complexity or limited commercial incentives. Compared with other countries, Switzerland’s timelines remain relatively long, although broadly similar to those in other publicly funded systems [17, 19]. Since 2011, ODs not included in the LS may still be reimbursed under Article 71a–c of the Ordinance on Health Insurance (OAMal), provided certain criteria are met (Fig. 1).

This study aimed to evaluate access to ODs in adult patients with IMDs treated at a Swiss reference center and to analyze the relevant regulatory and reimbursement frameworks at both national and international levels.

## Materials and methods

In this retrospective observational study conducted at the Adult Metabolic Clinic of Lausanne University Hospital between January 2017 and October 2022, we included all adult patients (aged ≥ 18 years) with confirmed biochemical and/or genetic diagnosis of an IMD as well as patients with selected rare genetic disorders outside the IMD classification who were treated with at least one OD during the study period. Patients with Fabry disease were not included in this study, as they were followed by a separate dedicated clinic at our institution. Patients with porphyria were also excluded, as access to therapy was coordinated by the Swiss Reference Center for Porphyrias in Zurich.

Overall, we collected patient-level clinical data as well as administrative data related to the process to obtain OD therapy. We also analyzed the regulatory and reimbursement frameworks relevant to the ODs used. The primary outcome included the total number of adult patients with IMDs or selected rare genetic disorders followed at our clinic during the study period and calculated the proportion who received at least one OD. The secondary outcomes included (1) the number and type of ODs used; (2) the proportion of ODs requiring special reimbursement procedures and the administrative process; (3) the time interval between formal treatment indication and initiation of OD therapy; and (4) the estimated yearly OD cost per patient, categorized into cost brackets. Additionally, we analyzed and compared national and international regulatory timelines, including the year of the first OD designation, the date of first MA in Switzerland and abroad (European Union, United States, Japan), and the timing of inclusion in the LS. International data were retrieved from the EMA, FDA, PMDA and Swissmedic for Switzerland.

The patients included in the study were classified based on their specific IMD and online Mendelian inheritance in Man (OMIM) number [4]. Electronic and paper patient records from the Divisions of Genetic Medicine were reviewed for diagnosis and confirmation (biochemical and/or molecular), and clinical features, including sex, age at diagnosis and at the start of treatment (only treatments with an OD designation, were analyzed). Treatments were classified as enzyme replacement therapy, substrate inhibitor, cofactor, chelator, bile acid replacement therapy or hormone therapy. Timelines between treatment indication and access to treatment were measured in months, starting from the initial request for coverage by the physician to the health insurance and ending with confirmation of coverage. The administrative process was quantified by the number of correspondence exchanges with health insurance. When specified by the insurance, the duration of coverage was reported in months. The estimated annual cost of each drug was calculated per patient via available pricing data from the Swiss Ordinance on the Services of Health Insurance (OPAS) [20], the LS [18] and publicly available pharmaceutical company data (either from official websites or direct inquiries). When multiple pricing sources existed, the ex-factory price (“prix fournisseur”) was used when available. For each patient, the cost estimate was based on the most recent year of follow-up and reflected the annualized cost of the latest prescribed OD regimen, taking into account the actual dose and frequency prescribed. Prices reflected market conditions as of 2022–2023, depending on the availability of pricing data. Costs were presented in Swiss francs (CHF) throughout the manuscript. For international comparability, equivalent amounts in euros (EUR) were estimated using an average 2022–2023 exchange rate of 1 CHF ≈ 1.01 EUR.

Clinical and treatment-related data were entered into a structured Microsoft Excel spreadsheet designed for the study. Administrative data, including insurance coverage requests, correspondence with insurers, and reimbursement decisions, were also collected and cross-checked for completeness. To ensure data accuracy and consistency, two independent investigators (GDA and CT) reviewed all the entries, and any discrepancies were resolved through consensus with a third investigator (CK). Internal consistency checks were performed to validate the dataset, including chronological verification of key dates (e.g., diagnosis and treatment initiation) and cross-checking of diagnostic and treatment classifications. Descriptive analyses were conducted via Microsoft Excel (Office 365, Version 2502).

This retrospective study was approved by the Swiss Ethics Committee on Research involving Humans (Approval No 2022–01470) and was conducted in accordance with predefined objectives and methodologies. The study was registered on ClinicalTrials.gov (Identifier: NCT05818566) on April 18th 2023. Patients provided written General Consent or documented oral consent after study information, both approved by the competent ethics committee. Oral consent complied with Article 9 of the Swiss Human Research Ordinance. 

### Statistical analysis

Descriptive statistical analyses were performed using Microsoft Excel statistical functions.

Prevalence was reported as a percentage of the total group or relevant subgroup, depending on the outcome studied. Continuous variables, including age and time intervals (e.g., delays between treatment indication and initiation, regulatory approval timelines, and reimbursement procedures), were summarized as median with interquartile range (IQR) and range (minimum–maximum), calculated using the inclusive quartile method (P25–P75). Categorical variables were presented as counts and percentages.

## Results

### Cohort overview

**Fig. 2:**
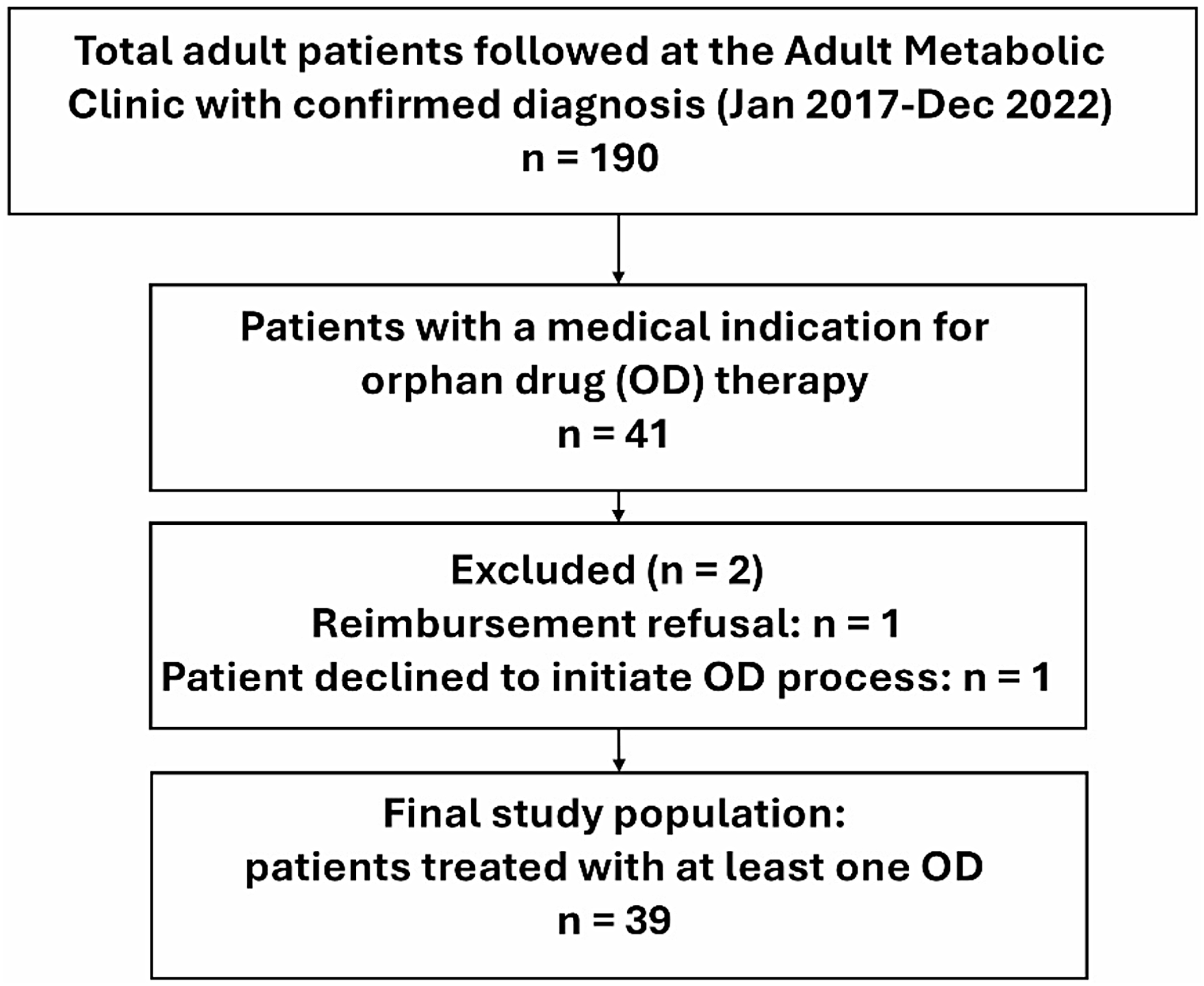
Study flowchart. Among the 190 adult patients followed at our center between 2017 and 2022, 41 had a medical indication for orphan drug (OD) therapy. Among these patients, 39 received an OD and were included in the study. Two patients were excluded: one due to reimbursement refusal by health insurance and one who declined to initiate therapy

The patient selection chart is shown in Fig. 2. A total of 190 patients were followed in our clinic at the time of analysis between 2017 and 2022. Among these patients, 100 (52.6%) had small molecule disorders, 55 (28.9%) had energy defect disorders, 26 (13.7%) had complex molecule disorders, 5 (2.6%) had non-IMD disorders, and 4 (2.1%) had bile acid synthesis disorders. Eight patients (4.2%) lost to follow-up. In addition to ODs, other metabolic treatments (e.g., cofactors, metabolite scavengers, and vitamin supplementation) were used in 81 patients (42.6%), 33 patients (17.4%) had no specific treatment, and dietary support (modified diets or nutrient supplementation, e.g., amino acid mixtures) was provided to 28 patients (14.7%). One patient with X-linked adrenoleukodystrophy died during follow-up.

### OD-treated patients

Among the 41 of 190 patients with an indication for OD therapy, 39 (20.5%) received an OD and were included in the study. This corresponds to treatment initiation in 95.1% (39/41) of patients with an indication for OD therapy. Patient characteristics are summarized in Table 1. Twenty-two patients were men, and 17 were women. Median age at analysis was 30.3 years (IQR 24.7–36.8; range 19–65). Among the 39 patients, 21 (53.8%) had small-molecule disorders, 13 (33.3%) complex-molecule disorders, 3 (7.7%) bile acid synthesis disorders (cerebrotendinous xanthomatosis), 1 (2.6%) glycerolipid metabolism disorder (Berardinelli–Seip lipodystrophy), and 1 (2.6%) non-IMD disorder (Werner syndrome). Median age at diagnosis was 11 years (IQR 0–32; range: 0–61), with 56.4% (22/39) diagnosed before age 20. Among the 39 patients, 43.6% (17/39) started OD treatment during infancy, and 56.4% (22/39) initiated therapy in adulthood (≥ 20 years, per Swiss Invalidity Insurance definition).

**Table 1:** Disorders by category, OMIM code and proportion of treated patients (*N* = 39)

	OMIM#	*n*	(%) of Total (*N* = 39)
**Small Molecules Disorders**			
Phenylketonuria	261600	8	20.5
Wilson Disease	277900	4	10.3
Ornithine Transcarbamylase Deficiency	311250	1	2.6
Alkaptonuria	203500	1	2.6
Classical homocystinuria	236200	3	7.7
Cobalamin C deficiency	277400	4	10.3
**Complex Molecules Degradation Disorders**			
Pompe Disease	621314	6	15.4
Gaucher Disease type I	77259	3	7.7
MPS IVA (Morquio Syndrome)	253000	1	2.6
MPS II (Hunter Syndrome)	309900	1	2.6
Niemann-Pick type B	607616	1	2.6
Niemann-Pick type C	257220	1	2.6
**Bile Acid Synthesis Disorder**			
Cerebrotendinous Xanthomatosis	213700	3	7.7
**Glycerolipid Metabolism**			
Berardinelli-Seip Lipodystrophy	269700	1	2.6
**Selected Other Rare Genetic Disorders**			
Werner Syndrome	277700	1	2.6
**Total**		**39**	**100**

OMIM: Online Mendelian Inheritance in Man; MPS: Mucopolysaccharidosis

Note: All diagnoses were confirmed biochemically and/or genetically

### OD therapies and treatment exposure

Seventeen different ODs were used: eight ERTs, two chelators, three substrate inhibitors, one cofactor, one hormone replacement therapy, one bile acid replacement therapy, and one methylating agent. All medically indicated patients received OD treatment, except for two: one with cerebrotendinous xanthomatosis whose insurance denied chenodeoxycholic acid (Leadiant^®^) despite multiple appeals, and one with Niemann–Pick disease type B who declined ERT.

Three patients were the first adults in Switzerland to receive olipudase alfa (Niemann–Pick type B), pegvaliase (phenylketonuria), and metreleptin (Werner syndrome). One patient with alkaptonuria was the first to receive the newly authorized formulation of nitisinone after Swiss MA.

Side effects occurred in five patients: hypertension, tachycardia, or mild allergic reaction with alglucosidase alfa; diarrhea and weight loss with miglustat; and headache/drowsiness after sodium benzoate overdose. Treatment interruption occurred in 9/39 patients (23.1%) due to side effects (3/9), insurance limitations (1/9), drug shortages (1/9), psychological stress (1/9), or nonadherence (3/9). One patient had to return to France to continue sapropterin therapy due to Swiss insurance restrictions, resuming in Switzerland only two years later.

### OD reimbursement procedures

Seventeen different ODs were prescribed, eight of which were not regularly reimbursed in Switzerland and required special access procedures. Among these, 8/17 (47.1%) were not listed in the LS and required special access procedures. Overall, 56.4% (22/39) of patients received an OD listed in the LS or LSIC and were reimbursed following an initial request. For the remaining 43.6% (17/39), additional administrative steps were needed under Art. 71a–c OAMal. In 10.3% (4/39) of the cases, OD was authorized but not listed in the LS (Art. 71b OAMal). For 33.3% (13/39) of cases, OD held no Swiss MA but was authorized abroad (Art. 71c OAMal). No compassionate use program was applied during the study period.

Overall median time from physician request to insurance decision was 1 month (IQR 0.6–2.6; range 0.2–12.6), with variability across products. For patients requiring an OD listed in the LS only (15/39) or LSIC (5/39), median access time was 0.9 months (IQR 0.6–1.7; range 0.2–3.2) and 1 month (IQR 0.3–1.0; range 0.2–4.9), respectively. For ODs authorized by Swissmedic but not included in the LS (Art. 71b OAMal; 4/39), the median access time was 0.6 months (IQR 0.6–1.6; range: 0.5–4.4 months). In cases where the OD had no Swiss MA but was authorized abroad (Art. 71c OAMal; 7/39), the access delay was longer, with a median of 6.5 months (IQR 2.5–11.9; range 1.0-12.6 months). Data was not available for 8 patients. No cases of off-label or off-limitation use were recorded in this cohort (Art. 71a OAMal). Once coverage was approved, its duration varied among patients. Temporary approvals (3–12 months) occurred in 61.5% (24/39), requiring renewals whereas no time limitation was requested for the remaining patients (38.5%). In 56.4% of cases, multiple exchanges with insurers, including submission of medical justifications and additional scientific documentation by the physicians, were required, particularly for off-LS ODs (92.9% vs. 36.4% for LS-listed ODs; Additional File 1).

### Regulatory timelines and orphan designation

In addition to patient-level outcomes, we conducted a drug-level analysis of the regulatory and reimbursement timelines for each OD prescribed in the cohort. The interval between first orphan designation (FDA, EMA, PMDA) and Swiss designation ranged from 11 months to 25 years; six ODs still lack Swiss designation (Table 2). Swiss MA holders are detailed in Additional File 2. In our analysis, Swissmedic MA occurred a median of 1.9 years after other regulators approval (IQR 1.5–2.7; range 0.3–5.5). Among 17 ODs, 7 were listed in the LS and 1 in LSIC. Median interval between Swiss MA and LS inclusion was 1.1 years (IQR 0.5–3.4; range − 0.8–11.7). Median interval from the first international MA (EU, US or Japan) to Swiss LS inclusion was 3.0 years (IQR 1.4–6.5; range 0.7–16.5; Table 2).

**Table 2:** Characteristics of orphan drugs used in the cohort (*n* = 17)

Brand Name (CH/US*)	Active Substance	First Orphan Designation (RA)	First MA (RA)	Swiss Orphan Designation	Delay (Yrs)^β^	Switzerland MA	Delay (Yrs)^α^	LS Inclusion	Delay^§^ (Yrs)
**Enzyme Replacement Therapies**									
Cerezyme^®^	Imiglucerase	1991 (FDA)	1994 (FDA)	NA	NA	1999	4.8	2010	11.7
Elaprase^®^	Idursulfase	2001 (FDA)	2006 (FDA)	2006	4.9	2007	0.7	NA	NA
Myozyme^®^	Alglucosidase alfa	1997 (FDA)	2006 (EMA)	2006	9.2	2008	2.2	2011	3.5
Nexviadyme^®^	Avalglucosidase alfa	2013 (FDA)	2021 (FDA)	2020	6.4	2021	0.3	2023	1.1
Palynziq^®^*	Pegvaliase	1995 (FDA)	2018 (FDA)	NA	NA	NA	NA	NA	NA
Vimizim^®^	Elosulfase alfa	2009 (FDA)	2014 (FDA)	NA	NA	2016	1.9	NA	NA
Vpriv^®^	Velaglucerase alfa	2009 (FDA)	2010 (FDA)	2010	0.9	2011	1.5	2010	-0.8
Xenpozyme^®^	Olipudase alfa	2000 (FDA)	2022 (EMA)	2019	18.5	2023	1.5	NA	NA
**Substrate Inhibitors**									
Cerdelga^®^	Eliglustat	2008 (FDA)	2014 (FDA)	2018	10.5	2020	5.5	2022	2.3
Nityr^®^	Nitisinone	1995 (FDA)	2017 (EMA)	2020	25.2	2022	4.5	NA	NA
Zavesca^®^	Miglustat	1998 (FDA)	2002 (EMA)	2007	8.9	2004	1.9	2005	0.9
**Chelators**									
Triogen^®^	Trientine dihydrochloride	2016 (FDA)	2017 (EMA)	2019	2.5	2020	2.7	2020	0.3
N/A**	Sodium Benzoate	2019 (EMA)	2005 (FDA)	NA	NA	NA	NA	NA	NA
**Bile Acid Replacement Therapy**									
Leadiant^®^	CDCA	2007 (FDA)	2017 (EMA)	2016	9.1	2018	1.2	NA	NA
**Methylating Agent**									
Cystadane^®^*	Betaine anhydrous	1994 (FDA)	1996 (FDA)	NA	NA	NA	NA	NA	NA
**Cofactors**									
Kuvan^®^	Sapropterin dihydrochloride	2004 (FDA)	2007 (FDA)	2007	3.4	2009	1.8	2023^¶^	NA
**Hormone Replacement Therapy**									
Myalepta^®^	Metreleptin	2001 (FDA)	2014 (FDA)	NA	NA	NA	NA	NA	NA

CH: Switzerland; CDCA: Chenodeoxycholic acid; FDA: Food and Drug Administration; EMA: European Medicines Agency; LS: List of Pharmaceutical Specialties; MA: Market Authorization; NA: Not Available (or pending at the time of analysis in 2023); RA: regulatory authority

* For medicines not marketed in Switzerland, the brand name in the USA is indicated

**Sodium benzoate was prepared by a local pharmacy without a commercial brand name (Grosse Apotheke Dr. G. Bichsel AG, Interlaken, Switzerland)

^**β**^ Delay between first orphan designation (EMA or FDA) and Swiss orphan designation (Swissmedic). For Trientine, dates reflect the modern marketed product line rather than earlier historical approvals of the active substance.

^α^ Delay between first MA (EMA or FDA) and Swiss MA (Swissmedic)

^**§**^ Delay between first Swiss MA and inclusion in the LS (Swiss Federal Office of Public Health)

^¶^ In 2023, Dipharma SA launched a generic equivalent to Kuvan^®^, known as Sapropterin Dipharma^®^, in Switzerland and other European markets. Sapropterin dihydrochloride products from Dipharma SA were subsequently included in the LS in 2023

# The negative value (–0.8 years) indicates one instance where LS inclusion preceded Swissmedic authorization

Note: This table summarizes regulatory and reimbursement timelines for orphan drugs prescribed in the study cohort. The delay values represent the time lag in years between regulatory milestones. Swissmedic grants orphan designation and marketing authorization in Switzerland, whereas the Swiss Federal Office of Public Health regulates inclusion in the LS ) for reimbursement. Inclusion in LS was assessed for the specific indication relevant to each treatment

Sources: Regulatory data were retrieved from public databases of the EMA, the FDA, the Pharmaceuticals and Medical Devices Agency (PMDA, Japan), Swissmedic, and the LS (Swiss Federal Office of Public Health) . The data were verified via official drug labels and company communications where necessary [12–14, 18]

### Annual cost estimates

Annual cost per patient was estimated for each of the 17 ODs. 59% of the patients received ODs with estimated annual costs of at least CHF 100,000/year, including several exceeding CHF 500,000(~ EUR 101,000–505,000; Table 3). Therapies with annual costs that could exceed  CHF 300,000/year (~ EUR 303,000) depending on product and patient-specific dosing, included ERTs, substrate inhibitor (eliglustat), hormone replacement therapy (metreleptin), and bile acid therapies (chenodeoxycholic acid). The highest annual treatment costs exceeded CHF 700,000 (~ EUR 707,000) for certain ERTs, particularly when dosing was weight-based. Drugs < CHF 10,000/year (~ EUR 10,100) included nitisinone (substrate inhibitor) and betaine (methylation agent). For other therapies, such as sapropterin and trientine, annual costs were variable due to weight-based dosing and dose adjustments. Overall, estimation of cumulative annual treatment expenditures for the cohort reached CHF 8,236,480 (~ EUR 8,318,845).

**Table 3:** Annual estimated cost per patient for orphan drugs (*n* = 39)

Price range (CHF)	Number of Patients (*n*)	Percentage (%)
< 10 000	2	5.1
10 000–50 000	10	25.6
50 000-100 000	4	10.3
100 000-300 000	11	28.2
300 000-500 000	9	23.1
> 500 000	3	7.7
Total	39	100.0

Note: Cost estimates are based on the most recent year of follow-up and reflect the annualized cost of the most recent prescribed OD regimen per patient. Data were obtained from the Swiss List of Specialties (LS), the Ordinance on the Services of Health Insurance (OPAS), and pharmaceutical company sources (websites or direct inquiries). Prices reflect data available from 2022–2023 and are reported using the ex-factory price (“prix fournisseur”) when available. All costs are presented in Swiss francs (CHF). For reference, euro (EUR) equivalents may be estimated using the 2022–2023 average exchange rate (1 CHF ≈ 1.01 EUR)

## Discussion

This study analyzed data from 39 patients with IMDs treated with ODs, illustrating real-world challenges faced by a specialized rare disease clinic, particularly regarding treatment access, reimbursement procedures, and administrative workload.

### Orphan drug policy and regulatory framework in Switzerland

Policies supporting ODs began with the U.S. Orphan Drug Act (1983), followed by Japan (1993) and the EU Regulation (2000), which introduced incentives for rare disease drug development [21, 22]. Despite progress, disparities in access persist across Europe [10, 23–30]. Switzerland, outside the EU, has developed its own framework. The first legal reference to ODs was introduced with the Therapeutic Products Act (2002), followed by Swissmedic’s orphan designation pathway in 2006 [31].

Our findings highlight variability in OD access timelines compared with other regions [32–34]. A 2022 report from the Center for Innovation in Regulatory Science ranked Swissmedic and the EMA behind agencies in Canada, Australia, and Japan in terms of approval timelines [35, 36]. Access is further complicated by Switzerland’s decentralized insurance system, particularly when ODs are not included in the LS as well as age-dependent rules. In these cases, Article 71 OAMal procedures require case-by-case decisions, often involving temporary approvals and repeated renewals [37, 38], nevertheless in several cases, rapid access was achieved through Article 71 procedures, indicating that this mechanism can facilitate timely treatment when standard reimbursement pathways are not yet available.

### Patterns of OD use in IMDs

21.6% of patients had an indication for an OD. Nearly all received treatment, except one who declined and another denied reimbursement. ERTs were the most frequently used ODs, consistent with reports highlighting their predominance in lysosomal storage disorders compared with defects of energy or carbohydrate metabolism [39, 40].

### Administrative and systemic barriers

Although access was achieved in most cases, several hurdles were noted. Over half of reimbursement requests required multiple exchanges with insurers, and nearly half (47.1%) of the orphan drugs used in this cohort were not listed in the LS at the time of analysis. Even LS-listed ODs often carried restriction clauses. Approvals were frequently time limited (3–12 months), necessitating repeated reassessments and creating a substantial administrative burden, often borne by physicians responsible for these processes [41–43]. Additional delays arose from prolonged price negotiations or company decisions not to pursue LS inclusion. For example, one patient with cerebrotendinous xanthomatosis in our cohort was unable to access chenodeoxycholic acid due to unresolved reimbursement negotiations. This case highlights how administrative and pricing factors may delay or prevent access even when an effective therapy exists.

Overall, access patterns in our cohort reflect underlying structural factors, including a fragmented insurance system, limited market incentives for manufacturers, and the inherent uncertainty of rare disease evidence.

### Costs of orphan drugs

While ODs offer clinical benefits, they also impose significant financial pressure [8, 10, 44, 45]. In our cohort, cumulative annual therapy costs reached several million Swiss francs, in line with reports of per-patient costs frequently exceeding several hundred thousand euros per year [26, 46]. Prices reflect small patient populations, research and development costs, and long-term monitoring requirements [45, 47–49]. Confidential rebate agreements between insurers and companies complicate the estimation of the true economic burden [50]. Whereas some large companies may derive substantial profits from OD legislation [26, 41], smaller enterprises often struggle with Swiss pricing and regulatory hurdles.

### Recent regulatory reforms (2024–2025)

Since January 2024, revised rules for reimbursement of medicines in individual cases (Art. 71 OAMal) were introduced to improve access and transparency [51]. However, for rare diseases, where evidence is limited, reimbursement decisions often require independent expert input (Art. 71d, al. 5), adding complexity rather than reducing it. Patients may still face practical barriers despite theoretical coverage [52], and required price reductions in certain cases may discourage some companies from seeking LS inclusion, reinforcing reliance on Art. 71 procedures.

### Collaboration between stakeholders for patients with rare diseases

National organizations and coordination structures such as the National Coordination for Rare Diseases (Kosek) [53], patient organizations including ProRaris [54], professional networks such as the Swiss Group of Inborn Errors of Metabolism (SGIEM) [55], and multi-stakeholder initiatives such as the Rare Disease Action Forum (RDAF) [56] illustrate Switzerland’s efforts to strengthen collaboration between patient groups, clinicians, and policy in shaping rare disease policy. Reference centers play a pivotal role in consolidating expertise and guiding patients through complex reimbursement processes. Despite these initiatives, coordination between clinical reference centers and regulatory or reimbursement authorities remains limited. Stakeholder discussions in Switzerland have raised concerns that recent changes to the health insurance reimbursement framework, particularly Article 71a–d, may affect access to therapies for rare diseases. These debates echo the barriers identified in our study and highlight the need to balance cost containment with equitable access to novel therapies.

### Strengths and limitations

The strengths of this study include systematic patient-level data collection and comparison with international authorization frameworks. Limitations include the single-center design, modest sample size, focus on adults, and exclusion of costly non-OD therapies. Moreover, clinical outcomes were not systematically assessed. In addition, dietary therapies, an essential component of treatment for many IMDs, were not analyzed specifically in this study. These products are regulated through distinct reimbursement pathways, which can create additional challenges for treatment coverage and access. Future research should therefore integrate patient-reported experiences, long-term health outcomes, and the broader regulatory context of metabolic therapies, including specialized dietary products.

## Conclusion

Our findings highlight both progress and persistent barriers in OD access in Switzerland. Most patients ultimately received treatment, and four were the first adults nationally to do so, underscoring the pioneering role of reference centers. Recent reimbursement reforms are promising, but further alignment with EU timelines, integration of rare disease networks, and simplification of reimbursement procedures are required. Sustained collaboration among clinicians, patients, regulators, and insurers will be essential to ensure equitable and timely OD access [57–63].

## Electronic Supplementary Material

Below is the link to the electronic supplementary material.

Supplementary Material 1

Supplementary Material 2

## Data Availability

The datasets generated and/or analysed during the current study are not publicly available due to maintaining participant confidentiality and anonymity but are available from the corresponding author on reasonable request.
